# *In Vitro* Antibacterial Activity of Propyl-Propane-Thiosulfinate and Propyl-Propane-Thiosulfonate Derived from* Allium* spp. against Gram-Negative and Gram-Positive Multidrug-Resistant Bacteria Isolated from Human Samples

**DOI:** 10.1155/2018/7861207

**Published:** 2018-09-17

**Authors:** Antonio Sorlozano-Puerto, Maria Albertuz-Crespo, Isaac Lopez-Machado, Juan Jose Ariza-Romero, Alberto Baños-Arjona, Manuela Exposito-Ruiz, Jose Gutierrez-Fernandez

**Affiliations:** ^1^Department of Microbiology, School of Medicine and PhD Program in Clinical Medicine and Public Health, University of Granada-ibs, Granada, Spain; ^2^Department of Microbiology and Biotechnology, DMC Research Center, Granada, Spain; ^3^Unit of Methodology of Research and Biostatistics, Virgen de las Nieves University Hospital-ibs, Granada, Spain; ^4^Laboratory of Microbiology, Virgen de las Nieves University Hospital-ibs, Granada, Spain

## Abstract

**Background:**

The aim of this study was to compare the* in vitro* antibacterial activity of two compounds derived from* Alliaceae*, PTS (propyl-propane-thiosulfinate), and PTSO (propyl-propane-thiosulfonate), with that of other antibiotics commonly used against bacteria isolated from humans.

**Materials and Methods:**

A total of 212 gram-negative bacilli and 267 gram-positive cocci isolated from human clinical samples and resistant to at least one group of antibiotics were selected. In order to determine the minimum inhibitory concentration (MIC) and minimum bactericidal concentration (MBC) to various antibiotics as well as PTS and PTSO, all isolates underwent broth microdilution assay.

**Results:**

PTS showed moderate activity against* Enterobacteriaceae *with MIC_50_ (and MBC_50_) and MIC_90_ (and MBC_90_) values of 256-512 mg/L, while PTSO showed greater activity with MIC_50_ and MIC_90_ values of 64-128 mg/L and MBC_50_ and MBC_90_ values of 128-512 mg/L. These data show the bactericidal activity of both compounds and indicate that PTSO was more active than PTS against this group of bacteria. Both compounds showed lower activity against* P. aeruginosa* (MIC_50_ = 1024 mg/L, MIC_90_ = 2048 mg/L, MBC_50_ = 2048 mg/L, and MBC_90_ = 2048 mg/L, for PTS; MIC_50_ = 512 mg/L, MIC_90_ = 1024 mg/L, MBC_50_ = 512 mg/L, and MBC_90_ = 2048 mg/L, for PTSO) compared to those obtained in others nonfermenting gram-negative bacilli (MIC_50_ = 128 mg/L, MIC_90_ = 512 mg/L, MBC_50_ = 128 mg/L, and MBC_90_ = 512 mg/L, for PTS; MIC_50_ = 64 mg/L, MIC_90_ = 256 mg/L, MBC_50_ = 64 mg/L, and MBC_90_ = 256 mg/L, for PTSO) and also indicate the bactericidal activity of both compounds against these groups of bacteria. Finally, the activity against* S. aureus*,* E. faecalis*, and* S. agalactiae* was higher than that observed against enterobacteria, especially in the case of PTSO (MIC_50_ = 8 mg/L, MIC_90_ = 8 mg/L, MBC_50_ = 32 mg/L, and MBC_90_ = 64 mg/L, in* S. aureus*; MIC_50_ = 4 mg/L, MIC_90_ = 8 mg/L, MBC_50_ = 8 mg/L, and MBC_90_ = 16 mg/L, in* E. faecalis* and* S. agalactiae*).

**Conclusion:**

PTS and PTSO have a significant broad spectrum antibacterial activity against multiresistant bacteria isolated from human clinical samples. Preliminary results in present work provide basic and useful information for development and potential use of these compounds in the treatment of human infections.

## 1. Introduction

The use of conventional antibiotics for the prevention of infectious diseases and as growth promoters in animal production has fostered the appearance of resistant bacteria and the transmission of these pathogens to humans [1]. In addition, the use and sometimes misuse of antibiotics in humans has increased the occurrence of infections (urinary tract infections, respiratory tract infections, skin and soft tissue infections, etc.) caused by multiresistant bacteria, which has reduced the therapeutic options and has made necessary the selection of new molecules with antibacterial properties [2]. Natural compounds obtained from vegetables with antibacterial properties could be considered an alternative to conventional antibiotics [3].

In recent years, the antibacterial properties of some compounds obtained from* Allium* plants such as garlic (*Allium sativum*) and onion (*Allium cepa*) have been described. These can inhibit the growth of a range of gram-positive and gram-negative bacteria, including both pathogenic and commensal bacteria in humans and animals [4, 5].* Allium*-derived products have been reported to be effective even against those strains that have become resistant to antibiotics [6].

**Figure 1:**
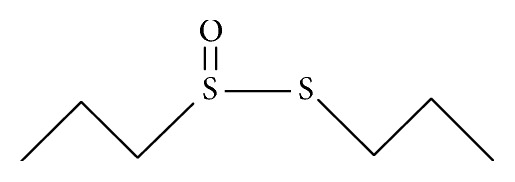
Chemical structure of propyl-propane-thiosulfinate (PTS).

**Figure 2:**
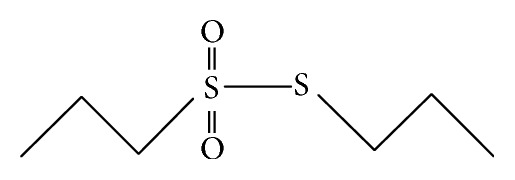
Chemical structure of propyl-propane-thiosulfonate (PTSO).

Two of these* Allium*-derived compounds, propyl-propane-thiosulfinate (PTS) (Figure 1) and propyl-propane-thiosulfonate (PTSO) (Figure 2), are organosulphurate products obtained by decomposition of initial compounds naturally present in garlic bulbs as alliin and allicin. In several* in vitro* and* in vivo* studies against pathogenic bacteria from animals, both compounds have showed an antibiotic activity [3, 7, 8]. While the precise mechanism of action is not yet known, the main antibacterial effect of thiosulfinates (as allicin) has been reported to be due to (i) its accessibility resulting from high permeability through phospholipid membranes [9]; (ii) its chemical reaction with thiol groups of various enzymes such as the bacterial acetyl-CoA-forming system, consisting of acetate kinase and phosphotransacetyl-CoA synthetase, blocking acetate incorporation into fatty acids and inhibiting the formation of lipids [10]; and (iii) the inhibition of RNA polymerase and RNA synthesis [11].

Therefore, the aim of this study was to compare the* in vitro* antibacterial activity of the compounds derived from garlic PTS and PTSO with that of other antibiotics commonly used against gram-negative and gram-positive multidrug-resistant bacteria isolated from human clinical samples.

## 2. Material and Methods

### 2.1. Antibiotics, PTS and PTSO

All antibiotics were purchased from Sigma-Aldrich (Madrid, Spain) and each antibiotic was dissolved according to the manufacturer's recommendations.

PTS and PTSO (95% purity) were supplied by DMC Research (Alhendín, Granada, Spain) and dissolved in polysorbate-80 to a final concentration of 50%. The biosynthesis of propyl-propane-thiosulfinate (PTS) and propyl-propane-thiosulfonate (PTSO) is made from propiin, an amino acid derived from L-cysteine found in* Allium* species. The first step of the biosynthesis is the formation of a sulfenic acid, which is highly reactive and immediately produces PTS by a condensation reaction. In the last step, oxidation of PTS induces its dismutation in PTSO and propyl disulfide that can be oxidized and transformed to PTSO and that way the oxidation of PTS to PTSO is completed.

### 2.2. Bacterial Isolates

A total of 212 gram-negative bacilli and 267 gram-positive cocci isolated from clinical samples obtained from 479 different patients were selected. Identification and susceptibility studies were performed using WIDER system (Francisco Soria Melguizo, Madrid, Spain) or MicroScan system (Siemens Healthcare Diagnostics, Madrid, Spain). The susceptibility results obtained through these systems allowed the selection of isolates, based on the resistance presence to at least one group of antibiotics commonly used in the treatment of infections caused by these bacteria.

The presence of extended-spectrum beta-lactamase-producing* Enterobacteriaceae *(ESBL) was confirmed by the diffusion method with disks containing cefotaxime (30 *μ*g), cefotaxime/clavulanic acid (30/10 *μ*g), ceftazidime (30*μ*g), and ceftazidime/clavulanic acid (30/10*μ*g). The resistance to methicillin was confirmed using the Mueller–Hinton agar diffusion procedure with 30 *μ*g cefoxitin disks. Both procedures were performed as recommended by the Clinical and Laboratory Standards Institute [12].

A total of 151 clinical isolates of* Enterobacteriaceae* (68* Escherichia coli*, 33* Klebsiella pneumoniae*, 6* Klebsiella oxytoca*, 15* Salmonella* spp., 17* Yersinia enterocolitica*, 7* Enterobacter cloacae*, 2* Providencia stuartii*, 1* Citrobacter amalonaticus*, 1* Kluyvera cryocrescens*, and 1* Proteus vulgaris*), 61 of nonfermenting gram-negative bacilli (40* Pseudomonas aeruginosa*, 9* Acinetobacter baumannii*, 7* Aeromonas hydrophila*, 3* Stenotrophomonas maltophilia*, 1* Achromobacter xylosoxidans*, and 1* Comamonas acidovorans*), 112* Staphylococcus aureus* (all of them methicillin-resistant), 54* Enterococcus faecalis *(all of them fluoroquinolone-resistant), and 101* Streptococcus agalactiae* were selected. All isolates were stored at -40°C until the susceptibility study by microdilution.

### 2.3. In Vitro Antibacterial Assay

In order to determine the antibacterial susceptibilities, all 479 isolates underwent broth microdilution assay in Cation-Adjusted Mueller–Hinton Broth (CAMHB) following the guidelines of the CLSI [12]. Broth microdilution testing was performed with 96-well, round-bottom microtiter plates with a final concentration of the bacterial cell suspension equal to 1 x 10^5^ colony forming units per milliliter (CFU/ml) in each well.

Each plate included negative controls (medium only) and 11 serial twofold dilutions of each antibiotic, PTS, or PTSO. The positive controls (only bacterial suspension without antibiotics) were added per well in a separate round-bottom plate.

The concentration ranges (in mg/L) assayed for* Enterobacteriaceae *for each antibiotic were the following: amoxicillin/clavulanate (0.25/0.125-256/128), piperacillin/tazobactam (0.5/4-512/4), cefuroxime (0.5-512), cefoxitin (0.5-512), cefotaxime (0.125-128), ceftazidime (0.5-512), cefepime (0.25-256), imipenem (0.016-16), meropenem (0.016-16), gentamicin (0.125-128), tobramycin (0.125-128), amikacin (0.5-512), ciprofloxacin (0.125-128), trimethoprim/sulfamethoxazole (0.06/1.1875-64/1216), and nitrofurantoin (1-1024). The concentration ranges assayed for nonfermenting gram-negative bacilli for each antibiotic were piperacillin/tazobactam (0.5/4-512/4), ceftazidime (0.5-512), cefepime (0.25-256), imipenem (0.125-128), meropenem (0.125-128), gentamicin (0.125-128), tobramycin (0.125-128), amikacin (0.5-512), and ciprofloxacin (0.125-128). The concentration ranges for staphylococci were gentamicin (0.25-256), tobramycin (0.25-256), erythromycin (0.06-64), clindamycin (0.06-64), levofloxacin (0.06-64), linezolid (0.03-32), vancomycin (0.015-16), teicoplanin (0.03-32), daptomycin (0.008-8), rifampicin (0.03-32), and trimethoprim/sulfamethoxazole (0.06/1.1875-64/1216). The concentration ranges for enterococci were ampicillin (0.03-32), levofloxacin (0.06-64), linezolid (0.008-8), vancomycin (0.06-64), teicoplanin (0.03-32), and daptomycin (0.008-8). Finally, the concentration ranges assayed for* S. agalactiae* for each antibiotic were ampicillin (0.004-4), erythromycin (0.06-64), clindamycin (0.06-64), levofloxacin (0.06-64), linezolid (0.008-8), vancomycin (0.008-8), and daptomycin (0.008-8).

The concentration ranges of PTS were 2-2048 mg/L in* Enterobacteriaceae*, nonfermenting gram-negative bacilli and* S. aureus*, and 4-4096 mg/L in* E. faecalis* and* S. agalactiae*. For PTSO, they were 2-2048 mg/L in* Enterobacteriaceae* and nonfermenting gram-negative bacilli and 0.125-128 mg/L in gram-positive cocci. Thus, the final concentration of polysorbate-80 in the wells was less than 1%.

The minimum inhibitory concentration (MIC) was defined as the lowest antibiotic concentration to completely inhibit the visible growth of a microorganism after overnight incubation and the isolates were considered to be susceptible, intermediate, or resistant, according to the recommendations of the CLSI [12]. A “susceptible” result indicates that the patient's organism should respond to therapy with that antibiotic using the dosage recommended normally for that type of infection and species. Conversely, a microorganism with a MIC interpreted as “resistant” should not be inhibited by the concentrations of the antibiotic achieved with the dosages normally used with that drug. An “intermediate” result indicates that a microorganism falls into a range of susceptibility in which the MIC approaches or exceeds the level of antibiotic that can ordinarily be achieved and for which clinical response is likely to be less than with a susceptible strain. MIC_50_ and MIC_90_ values were defined as the lowest concentration of the antibiotic at which 50 and 90% of the isolates were inhibited, respectively.

For minimum bactericidal concentration (MBC) testing, 100 *μ*l of broth from 1 to 5 wells containing no growth (which showed no visible turbidity) was plated onto antibiotic-free Columbia agar and incubated overnight at 37°C. The highest dilution that yielded no single bacterial colony on the agar plates was taken as MBC. Allium extracts were then considered as bacteriostatic or bactericidal depending on the MBC/MIC ratio which were, respectively, greater than 2 or between 2 and 1. MBC_50_ and MBC_90_ values were defined as the concentration of the antibiotic which kills 50 and 90% of the isolates, respectively.

Following the CLSI guidelines, we used the following strains as quality control in the procedures:* E. coli* ATCC 25922,* P. aeruginosa* ATCC 27853,* S. aureus* ATCC 29213, and* E. faecalis* ATCC 29212.

### 2.4. Statistical Analysis

Data analysis was performed using the software IBM SPSS Statistics v19. The Mann–Whitney U test was used to compare the distribution of MIC and MBC values of PTS and PTSO in the different groups of bacteria studied. A level of significance was considered with a p< 0.05.

## 3. Results

Tables 1 and 2 show the values (in mg/L) of the MIC_50_, MIC_90_, MBC_50_, and MBC_90_ and percentages of resistance to the antibacterial agents tested of the 479 clinical isolates.

There was 59 ESBL-producing* Enterobacteriaceae* (42* E. coli*, 12* K. pneumoniae*, and 5* K. oxytoca*). The presence of this resistance phenotype in 39.1% of* Enterobacteriaceae* was the main determinant of the high rates of resistance to beta-lactam antibiotics, whose range oscillated from 1.3% to meropenem (MIC_50_ = 0.125 mg/L, MIC_90_ = 1 mg/L) to 81.5% to cefuroxime (MIC_50_ > 512 mg/L, MIC_90_ > 512 mg/L).

ESBL-producing strains were more resistant to second to fourth-generation cephalosporins, such as cefuroxime (MIC_50_ > 512 mg/L, MIC_90_ > 512 mg/L, 100% resistant), cefotaxime (MIC_50_ = 128 mg/L, MIC_90_ > 128 mg/L, and 100% resistant), ceftazidime (MIC_50_ = 64 mg/L, MIC_90_ = 256 mg/L, and 78.0% resistant), and cefepime (MIC_50_ = 32 mg/L, MIC_90_ = 128 mg/L, and 93.2% resistant) that combinations of beta-lactams with beta-lactamase inhibitors such as piperacillin-tazobactam (MIC_50_ = 8/4 mg/L, MIC_90_ = 256/4 mg/L, and 30.5% resistant) and amoxicillin/clavulanate (MIC_50_ = 16/8 mg/L, MIC_90_ > 256/128 mg/L, and 52.5% resistant) or to carbapenems such as imipenem (MIC_50_ = 0.5 mg/L, MIC_90_ = 1 mg/L, and 100% susceptible) or meropenem (MIC_50_ = 0.125 mg/L, MIC_90_ = 1 mg/L, and 100% susceptible). Nevertheless, the absence of ESBL in* Salmonella* spp. and* Yersinia* spp. explains the lower number of isolates resistant to beta-lactam antibiotics in this group of enterobacteria (range 0-28.1%). Finally, in case of bacteria such as* Enterobacter *spp.,* Proteus *spp., or* Providencia *spp., among others (*remaining enterobacteria *group in Table 1), high rates of resistance to beta-lactams were observed: 16.7% to meropenem (MIC_50_ = 0.06 mg/L, MIC_90_ = 1 mg/L) and 100% to cefuroxime (MIC_50_ > 512 mg/L, MIC_90_ > 512 mg/L).

Among the aminoglycosides, amikacin was the antibiotic with a higher rate of activity against enterobacteria (MIC_50_ = 16 mg/L, MIC_90_ > 512 mg/L, and 29.1% resistant), against 35.8% resistant to gentamicin (MIC_50_ = 4 mg/L, MIC_90_ = 128 mg/L) or 42.4% to tobramycin (MIC_50_ = 4 mg/L, MIC_90_ = 128 mg/L). The resistance to aminoglycosides was higher among* Klebsiella *spp. and the group “remaining enterobacteria” than* E. coli*,* Salmonella *spp., or* Yersinia *spp. In general, enterobacteria showed high resistance to fluoroquinolones (MIC_50_ = 64 mg/L, MIC_90_ > 128 mg/L, and 67.6% of resistant isolates to ciprofloxacin) and to trimethoprim-sulfamethoxazole (MIC_50_ = 2/38 mg/L, MIC_90_ > 64/1216 mg/L, and 53.0% resistant), except for* Salmonella* spp. and* Yersinia* spp., which showed the lowest rates (37.5% and 3.1% of resistant isolates to ciprofloxacin and trimethoprim-sulfamethoxazole, respectively).* E. coli* was the bacteria with a lower resistance to nitrofurantoin (MIC_50_ = 32 mg/L, MIC_90_ = 64 mg/L, and 14.7% resistant).

As previously mentioned, bacteria were selected for their detection of resistance to, at least, a group of antibiotics. However, a relevant characteristic of the 151 enterobacteria included in the study was the high frequency to coresistance to two or more of these groups (multidrug-resistant bacteria), as described in Table 3. Therefore, 74.0% of the isolates resistant to some beta-lactams antibiotics were also resistant to ciprofloxacin, 61.8% to trimethoprim-sulfamethoxazole, and 48.8% to some aminoglycoside. It should be noted that 22.8% of that resistant to beta-lactams was also resistant to all the other groups of antibiotics assayed.

The behaviour of PTS and PTSO against multidrug-resistant enterobacteria was quite homogeneous, regardless the group analyzed (Table 1). The values of MIC_50_ and MIC_90_ of PTS ranged from 128 to 256 mg/L and from 256 to 512 mg/L, while the MBC_50_ and MBC_90_ ranged from 256 mg/L and 256 to 512 mg/L, respectively. On the other hand, the values of MIC_50_ and MIC_90_ of PTSO ranged from 64 to 128 mg/L and 128 to 256 mg/L, while MBC_50_ y MBC_90_ ranged from 64 to 128 mg/L and from 128 to 512 mg/L, respectively. These data show the bactericidal activity of both compounds (MIC and MBC values were equal or differed in only one dilution) and indicate that PTSO was significantly more active than PTS against this group of bacteria (p<0.001).

Among the 61 nonfermenting gram-negative bacilli, the resistance to beta-lactams antibiotics ranged from 32.8% to ceftazidime (MIC_50_ = 8 mg/L, MIC_90_ = 128 mg/L) and 52.5% to imipenem (MIC_50_ = 16 mg/L, MIC_90_ = 128 mg/L) and meropenem (MIC_50_ = 4 mg/L, MIC_90_ = 64 mg/L). Carbapenems showed more activity against bacteria such as* Acinetobacter *spp.,* Aeromonas *spp., and* Stenotrophomonas *spp. (MIC_50_ = 2 mg/L, MIC_90_ > 128 mg/L, and 42.9% of isolates resistant to imipenem and MIC_50_ = 2 mg/L, MIC_90_ = 16 mg/L, and 42.9% of isolates resistant to meropenem), than against* Pseudomonas* spp. (MIC_50_ = 16 mg/L, MIC_90_ = 128 mg/L, and 57.5% of isolates resistant to imipenem and MIC_50_ = 4 mg/L, MIC_90_ = 64 mg/L, and 57.5% of isolates resistant to meropenem). Among the aminoglycosides assayed, amikacin was the most active against both groups (MIC_50_ = 8 mg/L, MIC_90_ = 128 mg/L, and 19.7% resistant). Finally, 59.0% of isolates were resistant to ciprofloxacin (MIC_50_ = 32 mg/L, MIC_90_ > 128 mg/L), which resulted in less active against* P. aeruginosa *isolates than against other bacteria of this group. As shown in Table 3, 75.0% of the isolates resistant to fluoroquinolones (ciprofloxacin) were also resistant to some beta-lactam antibiotic; 63.9% to some aminoglycoside and 55.6% showed resistance to these three groups of antibiotics.

Just as with the rest of antibiotics, when comparing the results obtained in* P. aeruginosa* with those obtained in others nonfermenting gram-negative bacilli, the behaviour, both of PTS and PTSO, was significantly different (Table 1). In the case of PTS, the results shown in* P. aeruginosa* were MIC_50_ = 1024 mg/L, MIC_90_ = 2048 mg/L, MBC_50_ = 2048 mg/L, and MBC_90_ = 2048 mg/L, while in the rest of bacteria they showed more activity (MIC_50_ = 128 mg/L, MIC_90_ = 512 mg/L, MBC_50_ = 128 mg/L, and MBC_90_ = 512 mg/L) (p < 0.001). Likewise, the results for PTSO indicated less activity against* Pseudomonas* spp. (MIC_50_ = 512 mg/L, MIC_90_ = 1024 mg/L, MBC_50_ = 512 mg/L, and MBC_90_ = 2048 mg/L) than against the rest of isolates (MIC_50_ = 64 mg/L, MIC_90_ = 256 mg/L, MBC_50_ = 64 mg/L, and MBC_90_ = 256 mg/L) (p < 0.001). In any case, these data also indicate the bactericidal activity of both compounds, especially PTSO that showed significantly more activity than PTS (p < 0.001).

Concerning the gram-positive cocci, all the isolates were susceptible to vancomycin, teicoplanin (*S. agalactiae *was not tested), daptomycin, and linezolid. Besides, all the isolates of* E. faecalis* and* S. agalactiae *were also susceptible to ampicillin (Table 2).

All the isolates of* S. aureus* were resistant to methicillin (this was the selection criteria in this bacteria) and therefore to all beta-lactams antibiotics. High rates of resistance to fluoroquinolones (MIC_50_ = 8 mg/L, MIC_90_ = 32 mg/L, 89.3% resistant to levofloxacin), to aminoglycosides (MIC_50_ = 64 mg/L, MIC_90_ > 256 mg/L, 79.5% resistant to tobramycin), to macrolides (MIC_50_ > 64 mg/L, MIC_90_ > 64 mg/L, 69.6% resistant to erythromycin), or to lincosamides (MIC_50_ > 64 mg/L, MIC_90_ > 64 mg/L, 49.1% resistant to clindamycin) were observed. In contrast, trimethoprim-sulfamethoxazole (MIC_50_ < 0.06 mg/L, MIC_90_ = 0.5 mg/L, 3.6% resistant) and rifampicin (MIC_50_ < 0.03 mg/L, MIC_90_ = 0.5 mg/L, and 3.6% resistant) showed the lowest rates of resistance. The 75.9% of these bacteria were resistant, both to aminoglycosides and fluoroquinolones, and 60.7% also showed resistance to macrolides and 45.5% also to clindamycin (Table 3). Finally, 100% of isolates of* E. faecalis* were resistant to levofloxacin (MIC_50_ = 32 mg/L, MIC_90_ = 64 mg/L) and resistance to any other antibiotic was not associated, whereas 86 out of 101 isolates of* S. agalactiae *were resistant to erythromycin and clindamycin.

PTSO showed significantly more activity than PTS in the three groups of gram-positive bacteria tested (p < 0.001, in all cases) and the values for MIC_50_, MIC_90_, MBC_50_, and MBC_90_ were, for both compounds, lower than those obtained against gram-negative bacteria (Table 2). However, MIC and MBC values in gram-positive bacteria differed significantly, especially for PTS (more than 2 dilutions), which indicates that these compounds could have a bacteriostatic but not a bactericidal effect against these bacteria at least at low concentrations.

## 4. Discussion

Organosulfur compounds obtained from* Allium* spp. such as PTS and PTSO have been proposed as an effective alternative to antibiotics to improve animal performance and prevent gastrointestinal disorders. This is due on the one hand to their greater stability in comparison to other natural compounds [13] and on the other hand to their activity against bacterial groups, such as* Enterobacteriaceae*,* Staphylococcus* spp.,* Enterococcus* spp.,* Clostridium* spp.,* Bacteroides* spp.,* Lactobacillus* spp.,* Bifidobacterium* spp., or* Campylobacter* spp., among others [3, 4, 7]. Furthermore, it has been shown that feed supplementation with these compounds improves the digestion and absorption of nutrients in the gastrointestinal tract by modulating the intestinal microbiota and increases the villus height and mucosal thickness [7, 8]. Beyond its use in animals, it is possible that these molecules may as well be useful in the human clinical practice, due to the fact that alliaceous plants have been traditionally used for their antibacterial, antioxidant, and cardiovascular properties, as has been known for centuries [6].

To our knowledge, this is the first study to evaluate the activity of PTS and PTSO against a selection of gram-negative and gram-positive multiresistant bacteria isolated from human clinical samples. Antibiotic susceptibility tests were performed in accordance with the procedure outlined by CLSI in order to determine if a bacterium is susceptible or resistant to each of the antibiotic assayed. Although the cut-off points for PTS or PTSO are unknown, perform the assay under the same conditions as the other antibiotics allow us to make comparisons with them.

Our results revealed that PTS showed moderate activity against* Enterobacteriaceae* with MIC_50_ (and MBC_50_) and MIC_90_ (and MBC_90_) values of 256-512 mg/L, while PTSO showed greater activity with MIC_50_ and MIC_90_ values of 64-128 mg/L and MBC_50_ and MBC_90_ values of 128-512 mg/L. These homogeneous results among the different groups of enterobacteria selected, regardless of the resistance shown to different antibiotics commonly used in clinical practice, reveal the bactericidal action of these compounds. According to these results, Ruiz et al. also proved a bactericidal effect against enterobacteria, such as* E. coli* and* Salmonella typhimurium *[3].

The activity against methicillin-resistant* S. aureus*,* E. faecalis*, and* S. agalactiae* was higher than that observed against enterobacteria, especially in the case of PTSO (MIC_50_ = 8 mg/L, MIC_90_ = 8 mg/L, MBC_50_ = 32 mg/L, MBC_90_ = 64 mg/L, in* S. aureus*; MIC_50_ = 4 mg/L, MIC_90_ = 8 mg/L, MBC_50_ = 8 mg/L, and MBC_90_ = 16 mg/L, in* E. faecalis* and* S. agalactiae*). The PTS activity against this group of bacteria was significantly lower, especially in the case of enterococci. Some authors have evaluated the potential of garlic allicin, a molecule structurally similar to PTS, to control oral pathogens, reporting inhibitory concentrations of 600 mg/L against* Streptococcus* spp. [14]. Other studies have reported a bacteriostatic effect of allicin against vancomycin resistant enterococci [15].

However, in contrast to the relatively good results obtained previously, both compounds showed lower activity against* P. aeruginosa *(MIC_50_ = 1024 mg/L, MIC_90_ = 2048 mg/L, MBC_50_ = 2048 mg/L, MBC_90_ = 2048 mg/L, for PTS; MIC_50_ = 512 mg/L, MIC_90_ = 1024 mg/L, MBC_50_ = 512 mg/L, and MBC_90_ = 2048 mg/L, for PTSO). It is possible that PTS and PTSO may be affected by active removal mechanisms when they come in contact with these bacteria. Further research is needed to determine with certainty the mechanisms involved in this increased resistance.

All these results are in agreement with the antibacterial effects of garlic previously described in the literature against bacterial isolates from animals and reference strains [3–6]. However, MBC determined in our experiment were much higher compared to Llana-Ruiz-Cabello et al. who demonstrated MBC lower than 5 mg/L in all cases [16]. The differences may be caused by different methodology.

In the present study, the values obtained for MIC and MBC in PTS and PTSO were very similar to those obtained in antibiotics such as nitrofurantoin, aminoglycosides, fluoroquinolones, and some beta-lactams. Based on the data obtained from MIC, the CLSI determines that a very large percentage of enterobacteria should be resistant to these antibiotics (as shown in Tables 1 and 2). It should therefore not be considered for clinical use. Likewise, we may think that the activity shown by PTS and PTSO should also not be considered for clinical use in humans considering the results obtained. However, due to the lack of susceptibility cut-off points for the compounds derived from garlic, no final conclusion can be drawn.

In correspondence with the need of discovering new potentially antibacterial natural products, the activity of these organosulfur compounds described in this study may be considered as promising. Furthermore, the use of naturally and potentially innocuous compounds that can be administered without high restrictions provided us with the possibility to discuss the viability of their application for the treatment of specific infectious pathologies, provided that adequate formulations are developed.

In our opinion, several therapeutic possibilities may be considered, i.e., superficial skin infections, such as acne, folliculitis or impetigo by topical use, the treatment of oral and gastrointestinal infections by oral administration, or even the treatment of urinary tract infections caused by multidrug-resistant bacteria applied by intravesical instillation (in the same way that colistin is used). The concentration of the substance in the source of the infection should always be high enough to guarantee that it exceeds the values of MIC against the bacteria causing these processes.

It is clear that, in order to evaluate the real effectiveness of these substances, either in this or another situation, further testing would be necessary with a more diverse and larger group of bacteria. Furthermore, it would be necessary to establish suitable administration routes for the compounds and its efficacy* in vivo*. Finally, the concentrations that they achieve in the different tissues and fluids would also need to be known.

Lastly, PTS and PTSO are perceived as harmless since these compounds occur naturally in foods such as garlic or onion. Nevertheless, further studies on pharmacokinetic and toxicological characteristics are required before safe clinical use is considered. Some recent studies on cell lines and experimental animals reported low acute and subchronic oral toxicity in PTSO and a lack of genotoxicity, both* in vitro* and* in vivo* models [16–19].

## 5. Conclusion

Our results demonstrate that PTS, but mainly PTSO, have a significant broad spectrum antibacterial activity against a selection of gram-negative and gram-positive multiresistant bacteria isolated from human clinical samples. Further work is needed to demonstrate the effectiveness of these compounds* in vivo* models, although preliminary results in present work provide basic and useful information for development and its potential use in the treatment of human infections.

## Figures and Tables

**Table 1:** Activity *in vitro* of PTS, PTSO, and others antibacterial agents against gram-negative organisms.

**Organisms (number of isolates)**	**MIC**_**50**_	**MIC**_**90**_	**MBC**_**50**_	**MBC**_**90**_	%** of resistant isolates**
	**(in mg/L)**	**(in mg/L)**	**(in mg/L)**	**(in mg/L)**	
***Enterobacteriaceae* (n=151)**					
Amoxicillin/clavulanate	32/16	256/128	64/32	256/128	59.6
Piperacillin/tazobactam	8/4	256/4	32/4	512/4	31.8
Cefuroxime	>512	>512	>512	>512	81.5
Cefoxitin	8	256	64	512	37.1
Cefotaxime	64	>128	128	>128	77.5
Ceftazidime	16	256	64	512	58.3
Cefepime	8	128	32	256	65.6
Imipenem	1	1	2	16	2.0
Meropenem	0.125	1	0.25	4	1.3
Gentamicin	4	128	32	>128	35.8
Tobramycin	4	128	32	>128	42.4
Amikacin	16	>512	64	>512	29.1
Ciprofloxacin	64	>128	128	>128	67.6
Trimethoprim/sulfamethoxazole	2/38	>64/1216	64/1216	>64/1216	53.0
Nitrofurantoín	32	256	128	512	43.0
PTS	256	512	256	512	-
PTSO	64	128	128	512	-
***Escherichia coli* (n=68)**					
Amoxicillin/clavulanate	16/8	256/128	64/32	>256/128	55.9
Piperacillin/tazobactam	8/4	128/4	32/4	256/4	26.5
Cefuroxime	>512	>512	>512	>512	95.6
Cefoxitin	8	128	32	256	33.8
Cefotaxime	128	>128	>128	>128	94.1
Ceftazidime	32	256	64	>512	75.0
Cefepime	16	128	64	256	80.9
Imipenem	0.5	1	2	4	0.0
Meropenem	0.06	1	0.125	4	0.0
Gentamicin	4	64	16	128	30.9
Tobramycin	4	64	16	128	33.8
Amikacin	8	32	32	128	14.7
Ciprofloxacin	64	128	128	>128	73.5
Trimethoprim/sulfamethoxazole	>64/1216	>64/1216	>64/1216	>64/1216	61.8
Nitrofurantoin	32	64	64	256	14.7
PTS	128	256	256	512	-
PTSO	64	128	128	512	-
***Klebsiella* spp. (n=39)**					
Amoxicillin/clavulanate	32/16	64/32	64/32	256/128	82.1
Piperacillin/tazobactam	32/4	512/4	64/4	>512/4	59.0
Cefuroxime	>512	>512	>512	>512	97.4
Cefoxitin	32	512	64	>512	53.8
Cefotaxime	64	>128	128	>128	97.4
Ceftazidime	64	256	128	512	76.9
Cefepime	16	64	32	256	79.5
Imipenem	1	1	2	4	0.0
Meropenem	0.06	0.25	0.125	1	0.0
Gentamicin	64	>128	64	>128	66.7
Tobramycin	32	>128	32	>128	76.9
Amikacin	64	>512	128	>512	53.8
Ciprofloxacin	128	>128	>128	>128	87.2
Trimethoprim/sulfamethoxazole	>64/1216	>64/1216	>64/1216	>64/1216	79.5
Nitrofurantoin	64	128	128	256	61.5
PTS	256	512	256	512	-
PTSO	128	256	128	512	-
**ESBL-producers (n=59)**					
Amoxicillin/clavulanate	16/8	>256/128	64/32	>256/128	52.5
Piperacillin/tazobactam	8/4	256/4	32/4	512/4	30.5
Cefuroxime	>512	>512	>512	>512	100
Cefoxitin	8	64	32	128	25.4
Cefotaxime	128	>128	>128	>128	100
Ceftazidime	64	256	256	>512	78.0
Cefepime	32	128	64	>256	93.2
Imipenem	0.5	1	2	4	0.0
Meropenem	0.125	1	0.25	4	0.0
Gentamicin	4	128	16	>128	37.3
Tobramycin	4	128	32	>128	47.5
Amikacin	16	128	64	128	25.4
Ciprofloxacin	64	>128	128	>128	74.6
Trimethoprim/sulfamethoxazole	>64/1216	>64/1216	>64/1216	>64/1216	62.7
Nitrofurantoin	32	128	64	256	32.2
PTS	128	256	256	512	-
PTSO	64	128	128	512	-
***Salmonella* spp. and *Yersinia* spp. (n=32)**					
Amoxicillin/clavulanate	8/4	256/128	64/32	256/128	28.1
Piperacillin/tazobactam	2/4	128/4	16/4	128/4	12.5
Cefuroxime	4	>512	32	>512	25.0
Cefoxitin	8	64	64	128	12.5
Cefotaxime	1	1	8	64	12.5
Ceftazidime	4	4	16	64	0.0
Cefepime	2	32	16	64	0.0
Imipenem	1	1	16	16	0.0
Meropenem	1	1	4	8	0.0
Gentamicin	4	4	32	32	3.1
Tobramycin	4	4	32	32	6.3
Amikacin	16	128	128	256	34.4
Ciprofloxacin	1	128	8	128	37.5
Trimethoprim/sulfamethoxazole	2/38	2/38	16/304	>64/1216	3.1
Nitrofurantoin	256	512	512	1024	65.6
PTS	256	256	256	512	-
PTSO	64	128	64	128	-
**Remaining enterobacteria (n=12)**					
Amoxicillin/clavulanate	64/32	128/64	256/128	256/128	91.7
Piperacillin/tazobactam	4/4	64/4	8/4	256/4	25.0
Cefuroxime	>512	>512	>512	>512	100
Cefoxitin	256	>512	256	>512	66.7
Cefotaxime	64	>128	128	>128	91.7
Ceftazidime	8	128	32	512	58.3
Cefepime	8	64	8	256	66.7
Imipenem	1	4	2	16	25.0
Meropenem	0.06	1	0.125	4	16.7
Gentamicin	4	32	32	>128	50.0
Tobramycin	8	32	16	>128	58.3
Amikacin	4	256	8	>512	16.7
Ciprofloxacin	1	>128	8	>128	50.0
Trimethoprim/sulfamethoxazole	2/38	>64/1216	16/304	>64/1216	50.0
Nitrofurantoin	64	>1024	128	>1024	83.3
PTS	128	256	256	256	-
PTSO	64	128	128	256	-
**Nonfermenting gram-negative bacilli (n=61)**					
Piperacillin/tazobactam	16/4	512/4	128/4	512/4	34.4
Ceftazidime	8	128	64	512	32.8
Cefepime	8	32	64	256	42.6
Imipenem	16	128	32	>128	52.5
Meropenem	4	64	16	128	52.5
Gentamicin	4	>128	32	>128	39.3
Tobramycin	4	>128	32	>128	27.9
Amikacin	8	128	32	256	19.7
Ciprofloxacin	32	>128	64	>128	59.0
PTS	1024	2048	1024	2048	-
PTSO	256	1024	512	2048	-
***Pseudomonas aeruginosa* (n=40)**					
Piperacillin/tazobactam	16/4	256/4	128/4	256/4	25.0
Ceftazidime	8	64	64	256	27.5
Cefepime	8	32	64	128	32.5
Imipenem	16	128	32	128	57.5
Meropenem	4	64	32	128	57.5
Gentamicin	4	>128	16	>128	37.5
Tobramycin	4	128	16	>128	17.5
Amikacin	8	32	32	128	12.5
Ciprofloxacin	32	>128	64	>128	62.5
PTS	1024	2048	2048	2048	-
PTSO	512	1024	512	2048	-
**Remaining nonfermenting gram-negative bacilli (n=21)**					
Piperacillin/tazobactam	128/4	512/4	512/4	>512/4	52.4
Ceftazidime	8	128	64	512	42.9
Cefepime	16	64	128	256	61.9
Imipenem	2	>128	16	>128	42.9
Meropenem	2	16	8	>128	42.9
Gentamicin	4	>128	32	>128	42.9
Tobramycin	4	>128	32	>128	47.6
Amikacin	16	256	64	256	33.3
Ciprofloxacin	4	>128	32	>128	52.4
PTS	128	512	128	512	-
PTSO	64	256	64	256	-

MIC: minimum inhibitory concentration; MBC: minimum bactericidal concentration; % of resistant isolates: percentages of isolates intermediate or resistant according to the criteria published by the CLSI (2016).

**Table 2:** Activity *in vitro *of PTS, PTSO, and others antibacterial agents against gram-positive organisms.

**Organisms (number of isolates)**	**MIC**_**50**_	**MIC**_**90**_	**MBC**_**50**_	**MBC**_**90**_	%** of resistant isolates**
	**(in mg/L)**	**(in mg/L)**	**(in mg/L)**	**(in mg/L)**	
***Staphylococcus aureus* methicillin-resistant (n=112)**					
Gentamicin	4	256	16	>256	48.2
Tobramycin	64	>256	>256	>256	79.5
Erythromycin	>64	>64	>64	>64	69.6
Clindamycin	>64	>64	>64	>64	49.1
Levofloxacin	8	32	32	>64	89.3
Linezolid	2	4	4	8	0.0
Vancomycin	0.5	1	1	4	0.0
Teicoplanin	0.25	1	0.5	4	0.0
Daptomycin	0.25	0.5	0.5	2	0.0
Rifampicin	≤0.03	0.5	0.125	1	3.6
Trimethoprim/sulfamethoxazole	≤0.06	0.5	0.5	2	3.6
PTS	64	128	512	1024	-
PTSO	8	8	32	64	-
***Enterococcus faecalis* (n=54)**					
Ampicillin	1	2	2	8	0.0
Levofloxacin	32	64	>64	>64	100
Linezolid	2	2	4	8	0.0
Vancomycin	0.5	1	2	4	0.0
Teicoplanin	≤0.03	0.125	0.25	1	0.0
Daptomycin	2	4	4	8	0.0
PTS	128	128	2048	4096	-
PTSO	4	8	8	16	-
***Streptococcus agalactiae* (n=101)**					
Ampicillin	0.06	0.125	0.125	0.5	0.0
Erythromycin	>64	>64	>64	>64	94.1
Clindamycin	>64	>64	>64	>64	85.1
Levofloxacin	0.5	1	2	8	6.9
Linezolid	1	2	2	4	0.0
Vancomycin	1	1	2	4	0.0
Daptomycin	0.125	0.5	0.5	2	0.0
PTS	64	128	512	2048	-
PTSO	4	8	8	16	-

MIC: minimum inhibitory concentration; MBC: minimum bactericidal concentration; % of resistant isolates: percentages of isolates intermediate or resistant according to the criteria published by the CLSI (2016).

**Table 3:** Analysis of coresistance to different groups of antibiotics.

	**Enterobacteria resistant to some beta-lactams antibiotics** **(n=123; 81.5**%**)**	**ESBL-producers enterobacteria** **(n=59; 39.1**%**)**	**Non ESBL-producers enterobacteria resistant to some beta-lactams antibiotics** **(n=64; 42.4**%**)**	**Non-fermenting gram-negative bacilli resistant to fluoroquinolones** **(n=36; 59.0**%**)**	**Methicillin-resistant *Staphylococcus aureus*** **(n=112)**
**Resistance to beta-lactams**	-	-	-	75.0%	-
**Resistance to aminoglycosides**	48.8%	47.5%	50%	63.9%	79.5%
**Resistance to fluoroquinolones**	74.0%	74.6%	73.4%	-	89.3%
**Resistance to TMX**	61.8%	62.7%	60.9%	-	3.6%
**Resistance to nitrofurantoin**	41.5%	32.2%	50.0%	-	-
**Resistance to macrolides (erythromycin)**	-	-	-	-	69.6%
**Resistance to lincosamides (clindamycin)**	-	-	-	-	49.1%
**Resistance to rifampicin**	-	-	-	-	3.6%
**Resistance to aminoglycosides & fluoroquinolones**	46.3%	44.1%	50.0%	-	75.9%
**Resistance to aminoglycosides & fluoroquinolones & TMX**	41.5%	39.0%	45.3%	-	2.7%
**Resistance to aminoglycosides & fluoroquinolones & TMX & nitrofurantoin**	22.8%	22.0%	23.4%	-	-
**Resistance to beta-lactams & aminoglycosides**	-	-	-	55.6%	-
**Resistance to aminoglycosides & fluoroquinolones & macrolides**	-	-	-	-	60.7%
**Resistance to aminoglycosides & fluoroquinolones & macrolides & lincosamides**	-	-	-	-	45.5%

TMX: Trimethoprim/sulfamethoxazole.

## Data Availability

The data used to support the findings of this study are available from the corresponding author upon request.
